# Sustainable Cement Composite Integrating Waste Cellulose Fibre: A Comprehensive Review

**DOI:** 10.3390/polym15030520

**Published:** 2023-01-19

**Authors:** Sarah Fernando, Chamila Gunasekara, Amin Shahpasandi, Kate Nguyen, Massoud Sofi, Sujeeva Setunge, Priyan Mendis, Md. Tareq Rahman

**Affiliations:** 1 School of Engineering, RMIT University, Melbourne, VIC 3000, Australia; 2 Department of Infrastructure Engineering, University of Melbourne, Melbourne, VIC 3000, Australia

**Keywords:** waste cellulose fibre, waste paper sludge, cement composite, mechanical properties, durability properties

## Abstract

This review presents the research conducted to date in the field of cement-based composites reinforced with waste paper-based cellulose fibres, focusing on their composition, mechanical properties, and durability characteristics. The literature demonstrates that the properties of raw material (depending on their own chemical composition) significantly influence the formation of the cement composite binders. When considering fresh properties, the presence of silica and magnesium compounds generally lead to favourable effects on the setting of the cement composite when combined with waste paper cellulose fibre. Reduction in density values, i.e., approximately 25%, was observed with the inclusion of waste paper fibres from 20 to 80% in cement composites. The homogeneous dispersion of fibres in the matrix is one of the crucial factors to achieve in order to develop composites with well-balanced mechanical properties incorporating waste paper cellulose fibres. Hence, dispersion of fibres can be improved by increasing water quantity corresponding to the optimal value, which was a water/cement ratio of 0.64 leading to optimum strength properties of the composite. Even though the effect of fibre dispersion in the matrix improves with the addition of water, higher porosity and voids govern the strength properties beyond an optimum water-to-cement ratio. Higher porosity leads to an increase in the water absorption and a lowering of the thermal conductivity properties with the addition of paper fibre in cement binders. Paper fibre absorbs a high amount of water leading to higher water absorption. This phenomenon is related to the hydrophilic nature of cellulosic fibres absorbing some volume of water due to their microporous structure.

## 1. Introduction

Management of waste has become an alarming issue around the world. Many countries are facing a serious challenge in disposing of waste nowadays. The current world population has been estimated to be 7.8 billion, and according to the United Nations (UN), it is predicted that the population will increase to 9.8 billion by the year 2050 [1]. This large population is using natural resources, and at the same time, landfills are filling up with waste products coming from different streams. Annually, the world generates around 2.01 billion tonnes of waste and 33 percent of that is not managed in an environmentally safe manner. Worldwide, there is around 0.74 kg of waste generation per person per day and it may change from 0.11 to 4.54 kg. Moreover, high-income countries generate around 34 percent of the world’s waste. Global waste generation is expected to increase to 3.40 billion tonnes by 2050 [2]. During the financial year 2018–2019, Australia produced 74 million tonnes of waste, a 10% increase since 2016–2017 [3]. Industries are coming together with researchers to find alternative ways to manage those wastes sustainably. Researchers are developing methods to reuse and recycle waste products to reduce landfill usage and close the loop of manufactured products to establish a circular economy. Various studies are being carried out in order to achieve some replacement of cement clinker with other industrial by-products, which offers benefits such as potential savings in natural resources and energy, reduction in impact of CO_2_ emission, and reuse of wastes. One such industrial by-product is waste paper/cardboard and waste paper sludge from paper manufacturing.

It is estimated that Australia’s annual paper and cardboard usage is 235 kg per person [3]. It was reported that during the financial year 2018–2019, Australia produced 5.92 million tonnes of paper and cardboard waste, where 1.9 million tonnes went directly to landfill. It is important to increase the recovery rate as an exporting ban on paper and cardboard waste will be effective by mid-2024 according to Table 1 [4]. According to the statistics, paper and cardboard account for around 26% of total waste at landfills globally [3]. From 2010 to 2060, the global consumption of pulp and paper is expected to double, and potentially this will increase the amount of paper and cardboard waste in landfills. After 60% of the current recovery rate, about 2.3 million tonnes of paper and cardboard waste are looking for new products or processes in order to be managed sustainably to reduce landfill usage in Australia.

**Figure 1:**
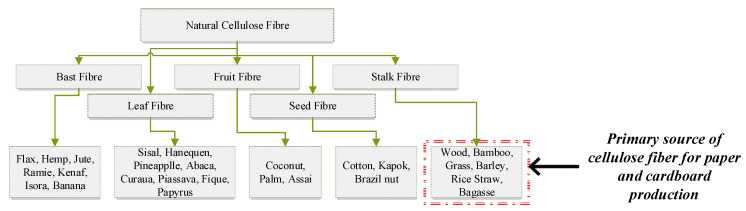
Classification of cellulose fibre.

The use of fibres in cement-based composite binders has been used for hundreds of years. Commonly used fibres in cement-based binders are steel, synthetic, and cellulose fibres, etc. Cellulose fibres used in concrete are eco-friendly, biodegradable, recyclable, and widely available throughout the world compared to other fibre types. Moreover, cellulose fibres exhibit interesting physical and mechanical properties, i.e., low density and well-balanced stiffness, toughness, and strength [5]. Cellulose fibres are mainly composed of cellulose with varying amount of lignin and hemicelluloses and other minority components (i.e., water, proteins, peptides, and inorganic compounds). Classification of natural cellulose fibre and the source of cellulose fibres in paper and cardboard production in Australia are illustrated in Figure 1.

Paper-based cellulose fibres used in cement-based composite are classified into two main categories: (1) waste paper sludge and (2) paper and cardboard waste. Waste paper sludge is also referred to as hypo-sludge [6]. Bajpai et al. [7] have reported that about 300 kg of dry sludge is generated in manufacturing for every one tonne of paper. This amount significantly varies within different regions, due to variation in recycling rates. The waste paper sludge consumes a large percentage of local landfill space each and every year. At present, this residual waste paper sludge is generally deposited in landfills causing disposal and environmental pollution problems. Some of the wastes are land spread on crop land as a disposal technique, raising concerns regarding trace contaminants (due to ink, dyes, coatings, pigments in the papers/cardboards) building up in soil or running off into the area’s lakes and streams. Hence, the utilisation of waste cellulose fibre can provide a sustainable solution and serve as an alternative supplementary material/fibre in cement-based composite binders.

Many studies have been performed using other recycled waste materials such as recycled coal bottom ash, waste lathe scraps, waste lathe fibres, and steel fibres extracted from waste tyres for cement-based composites [2,8,9,10,11,12,13,14] and these studies are also important as they address the reduction of environmental problems. In addition, studies have been published describing the use of waste cellulose-based fibres (i.e., both waste paper/cardboard and waste paper sludge) for cement-based composites [6,15,16,17,18,19,20,21]. The main objective of this review is to summarise the potential use of waste paper-based cellulose fibres as cementitious binder material for cement composite binders. Furthermore, a detailed review is provided on characterisation of waste cellulose fibre, activation mechanisms, pozzolanic reactivity, reaction kinetics, and its effect on mechanical and durability properties of cement-based composites.

## 2. Significance of Review

Published research on the use of paper-based waste cellulose fibre in concrete has not gained appreciable attention and limited research has been published (i.e., both waste paper/cardboard and waste paper sludge) for cement-based composites [6,15,16,17,18,19,20,21]. So far, no in-depth review has been undertaken based on the strength, microstructural, and durability properties of cement composite manufactured using paper sludge and waste paper/cardboard. The aim of this paper is to review cement composite binders incorporating paper-based waste cellulose fibres. Hence, this review aims at only the waste cellulose fibre in categories of the cellulose fibre classification. This paper will provide a systematic and comprehensive review on waste paper-based cement composites, focusing on the reaction mechanism and engineering performance of cement composite binders. The reviewed results, analysis, and discussion will be extremely useful to comprehend the behaviour of cellulose fibre in cementitious concrete.

## 3. Characterisation of Waste Paper Cellulose Fibre

Paper is a natural polymer which consists of wood cellulose. Papers are manufactured using virgin pulp from wood and non-wood sources or a combination of virgin pulp and recycled pulp composed of cellulose fibre sources from recycled papers. Depending on the treatment used to destroy or weaken the inter-fibre bonds, the pulping processes can be mechanical, thermal, chemical, or some combination of these treatments. Chemical pulping (kraft pulp) removes most of the lignin present originally in the wood whereas mechanical pulping processes leave most of the lignin in the fibres. Most of cellulosic pulps are obtained chemically, which breaks the bonds that link lignin, hemicellulose, and cellulose. Cellulose is made of units of monomer glucose. The chemical structure of cellulose (whose basic chemical formula is C_6_H_10_O_5_) comprises a long, repeating chain of carbon–hydrogen–oxygen units. Cellulose is water insoluble although it contains several hydroxyl groups. The physical–chemical properties of the paper-based waste cellulose fibres often depend on the pulping process, sources, cultivation and harvesting methods, and processing. The chemical and morphological properties reported for waste paper are presented in Table 2.

The composition of waste paper sludge varies largely depending on the grade of paper, raw material, processing technique, and quantity and quality of recycled paper used. Waste paper contains chemicals from a wide range of sources, such as additives, inks, dyes, pigments, and glues. Waste paper sludge produced at source has a high water content ranging from 40 to 70%. Elemental analysis shows that pozzolanic silica content in paper sludge was 60.5%. Pozzolanic silica participates in a pozzolanic reaction to form cementitious material. Elemental calcium content in paper water was 15%. Heavy metals copper (Cu), strontium (Sr), Zirconium (Zr), and manganese (Mn) were present in trace (less than 0.1%). Therefore, the possibility of leaching heavy metals is insignificant [20]. Researchers [19,20,23] carried out characterisation of waste paper sludge based on chemical composition, summarised in Table 3.

The thermogravimetric analysis (TGA) carried out by Rajput et al. [20] determined that 45% of mass loss occurred at 300 °C temperature. The first loss (7.5%) occurred between 30 °C and 280 °C, which is premature loss and could be attributed to the removal of superficial water molecules that may be present in the solid pores. The second mass loss occurs beyond 280 °C, where the material becomes thermally degraded and is sintered. The third mass loss beyond 300 °C is due to combustion of solid organic matter present in paper waste. It confirms that the phase change of paper waste takes place at 280 °C and it becomes thermally degraded. Based on the TGA curves, it can be concluded that the composite made from paper waste can withstand at the minimum of 300 °C. 

**Figure 2:**
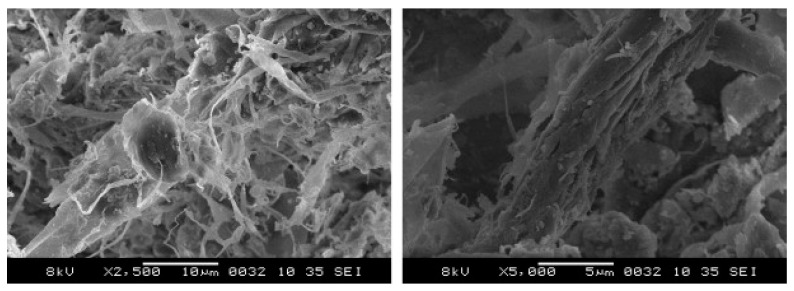
Microscopic view of raw waste paper used by Raut et al. [21] and Rajput et al. [20] for concrete brick production.

Raut et al. [21] and Rajput et al. [20] have looked into the microscopic condition of the paper waste and analysed the presence of irregular pores and fibre structures as presented in Figure 2. These hold the moisture in these pores and the fibrous envelopes provide an obstacle for moisture to move towards the surface. The fibrous nature provides a very high energy absorbing ability and high retention ability.

## 4. Waste Paper Sludge Cement Composite

The mix design and mechanical properties of waste paper-based cement composites are summarised in Table 4. Contrasting conclusions have been made by difference researchers on the effect of waste paper cellulose fibre replacement on mechanical properties. Raut et al. [21] and Rajput et al. [20] investigated cement (paste) brick samples prepared with an 80–95% of cellulose paper sludge replacement with cement, while other researchers [6,19,23,24] reported cement-based concrete composites with 5–40% of cement replaced with waste cellulose sludge. 

**Figure 3:**
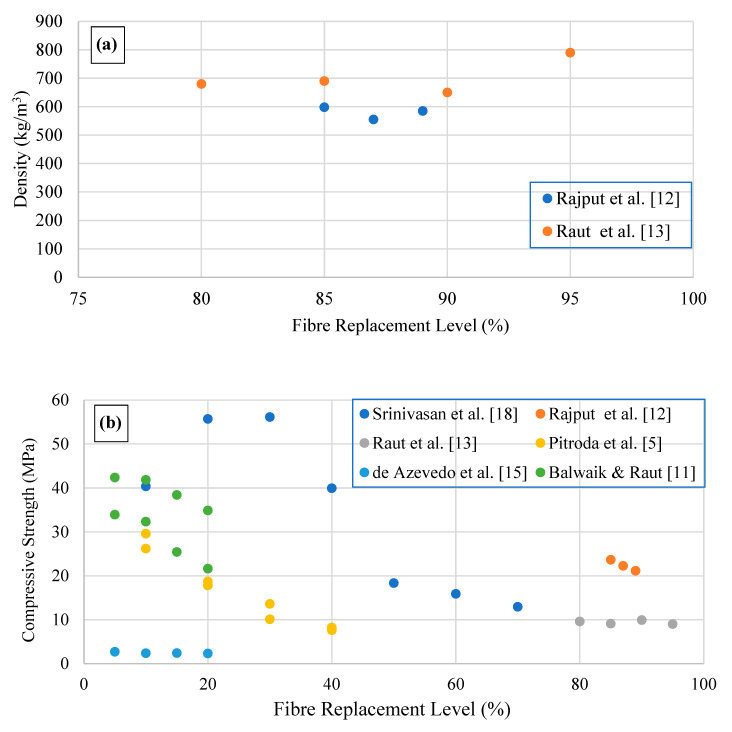

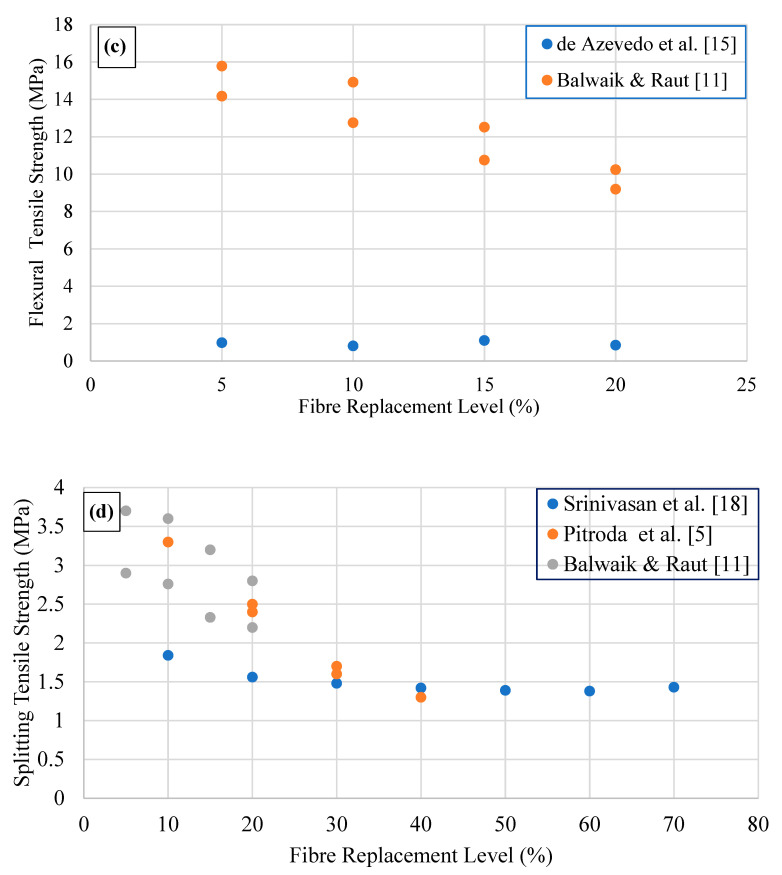
(**a**) Density; (**b**) 28-day compressive strength; (**c**) 28-day flexural tensile strength; and (**d**) 28-day splitting tensile strength variations of waste paper sludge cement composite binders.

The specific weight of brick samples (paste) prepared with cement (10% by weight) and cellulose paper sludge (89–85% by weight) waste ranges between 555 and 598 kg/m^3^ [20]. However, Raut et al. [21] reported a higher density range of 790–680 kg/m^3^ for 95–80% replacement of paper sludge, observing the lowest density value of 650–660 kg/m^3^ for 90–85% replacement of paper sludge for cement-based composite brick (paste) (Figure 3a). The bulk density of conventional cement or blended cement paste ranges between 1400 and 1800 kg/m^3^, which is much higher than blended cement pastes with waste cellulose fibre.

Rajput et al. [20] reported that cement brick samples prepared with 85, 87, and 89% cellulose paper waste successfully achieved compressive strengths of 23.64, 22.27, and 21.14 MPa, respectively. Moreover, Raut et al. [21] achieved the strength requirement as per ASTM C67 [25]: 9 ± 1 MPa which is three times higher than conventional clay bricks. During their investigation, the optimum cement and cellulose paper sludge content was determined as 10 and 90% by weight, achieving 9.9 MPa compressive strength, respectively. Seyyedalipour et al. [24] reported 28-day compressive strength increases up to 56.11 MPa with an increase of waste paper sludge percentage up to 30%, and further increments of waste paper sludge up to 70% resulted in a 77% decline in compressive strength of blended concrete. Furthermore, the addition of 10% waste paper sludge reduced the compressive strength, as reported by Pitroda et al. [6], of blended concrete by 34% (reduced by 15 MPa) when compared with control concrete specimens without incorporation of waste paper sludge. A drastic reduction was observed beyond 10% up to 40% (reduction by approximately 21 MPa) of waste paper sludge addition (Figure 3b). Balwaik and Raut [19] investigated the 28-day compressive strength of samples and found 40.7 MPa for 5% and 34.87 MPa for samples prepared with 20% of waste paper sludge.

Both splitting and flexural tensile strength reported by researchers showed a decreasing behaviour with waste paper sludge content (Figure 3c,d). Pitroda et al. [6] reported the splitting strength decline from 3.44 to 3.26 MPa and, from 3.96 to 3.59 MPa between 0 and 10% paper sludge content for 0.4 and 0.3 water/cement ratio, respectively. Further reductions of 63% (by 2.16 MPa) and 66% (2.63 MPa) were observed with up to 40% replacement of cement with waste paper sludge in cement-based concrete composites. A similar trend was observed by Srinivasan et al. [26] for splitting tensile strength with the incorporation of waste paper sludge in concrete from 0 to 50% (i.e., from 1.84 to 1.38 MPa).

**Figure 4:**
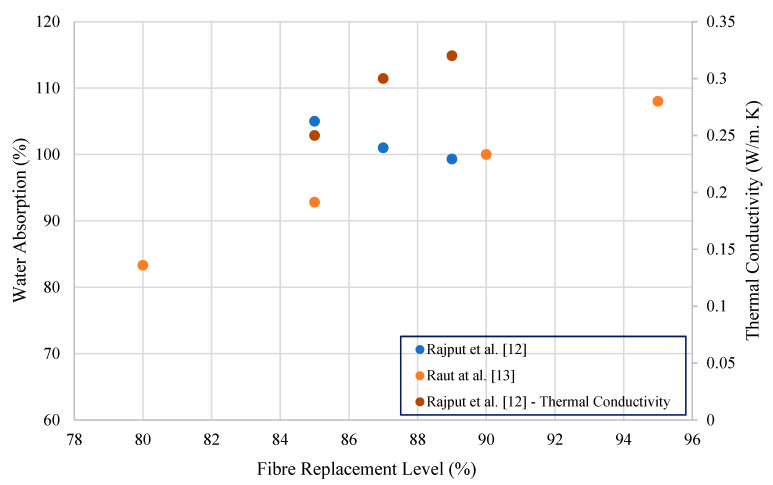
Water absorption and thermal conductivity variations of waste paper sludge cement composite binders.

The reported durability properties (i.e., water absorption and thermal conductivity) of waste paper sludge-based cement composites are summarised in Table 4 and illustrated in Figure 4. Raut et al. [21] observed that 95% replacement of waste paper sludge resulted in 108% water absorption in cement brick paste. From 95 to 80% replacement of cement with waste paper sludge, a decline in water absorption was shown, with approximately 23% reduction for cement brick paste. According to Rajput et al. [20], use of 85 to 89% of waste paper sludge in brick paste resulted in considerably higher water absorption (range between 105 and 99.3%) when compared with conventional clay brick and fly ash brick. Water absorption of burnt clay brick and fly ash brick was 14.2% and 14.6%, respectively [20]. Thermal conductivity of samples prepared by Rajput et al. [20] slightly increased from 0.25 W/mK (85% of paper sludge) to 0.32 W/mK (89% paper sludge).

**Table 4:** Mix design and performance of cellulose waste (waste paper and cardboard) cement-based composite.

Reference		Replacement Level (%)	Waste Cellulose Paper (wt %)	Cement (wt %)	Other (wt %)	Sand (wt %)	Coarse Aggregate (wt %)	Water (wt %)	Water/Cement	Density (kg/m^3^)	Compressive Strength (MPa)	Flexural Strength (MPa)	Splitting Tensile Strength (MPa)	Water Absorption (%)	Thermal Conductivity (W/mK)
[26]	Waste paper sludge	10	2.2	20.1	-	18.6	51.2	7.8	0.35	-	40.37	-	1.84	-	-
		20	4.5	17.9	-	18.6	51.2	7.8	0.35	-	55.69	-	1.56	-	-
		30	6.7	15.6	-	18.6	51.2	7.8	0.35	-	56.11	-	1.48	-	-
		40	8.9	13.4	-	18.6	51.2	7.8	0.35	-	39.95	-	1.42	-	-
		50	11.2	11.2	-	18.6	51.2	7.8	0.35	-	18.335	-	1.39	-	-
		60	13.4	8.9	-	18.6	51.2	7.8	0.35	-	15.87	-	1.38	-	-
		70	15.6	6.7	-	18.6	51.2	7.8	0.35	-	12.94	-	1.43	-	-
[20] Cotton waste	Paper sludge	85	23.8	2.8	1.4	-	-	72.0	-	598	23.64	-	-	105	0.25
		87	24.4	2.8	0.9	-	-	71.9	-	555	22.27	-	-	101	0.3
		89	25.0	2.8	0.3	-	-	71.9	-	585	21.14	-	-	99.3	0.32
[21]	Paper sludge	95	26	1	-	-	-	73	51.9	790	9.0	-	-	108.0	-
		90	24	3	-	-	-	73	27.4	650	9.9	-	-	100.0	-
		85	23	4	-	-	-	73	17.9	690	9.1	-	-	92.8	-
		80	22	5	-	-	-	73	13.6	680	9.6	-	-	83.3	-
[6]	Paper sludge	10	2.0	18.3	-	20.6	50.9	8.2	0.4	-	29.6	-	3.3	-	-
		20	4.1	16.3	-	20.6	50.9	8.2	0.4	-	17.8	-	2.4	-	-
		30	6.1	14.2	-	20.6	50.9	8.2	0.4	-	10.1	-	1.7	-	-
		40	8.1	12.2	-	20.6	50.9	8.2	0.4	-	8.2	-	1.3	-	-
		10	2.7	24.1	-	11.8	58.0	3.5	0.3	-	26.2	-	3.6	-	-
		20	5.3	21.4	-	11.8	58.0	3.5	0.3	-	18.7	-	2.5	-	-
		30	8.0	18.7	-	11.8	58.0	3.5	0.3	-	13.6	-	1.6	-	-
		40	10.7	16.0	-	11.8	58.0	3.5	0.3	-	7.6	-	1.3	-	-
[23] Lime	Waste paper sludge	5	0.5	10.4	9.9	62.3	9.9	17.0	1.6	-	2.69	0.98	-	-	-
		10	1.0	10.3	9.3	62.1	9.3	17.2	1.7	-	2.36	0.81	-	-	-
		15	1.5	10.3	8.8	61.9	8.8	17.5	1.7	-	2.38	1.1	-	-	-
		20	2.1	10.3	8.2	61.8	8.2	17.6	1.7	-	2.29	0.85	-	-	-
[19]	Waste paper sludge	5	0.8	15.5	-	23.4	52.1	8.2	0.5	-	33.93	14.17	2.9	-	-
		10	1.6	14.7	-	23.4	52.1	8.2	0.5	-	32.33	12.75	2.76	-	-
		15	2.5	13.9	-	23.4	52.1	8.2	0.5	-	25.43	10.75	2.33	-	-
		20	3.3	13.1	-	23.4	52.1	8.2	0.5	-	21.62	9.19	2.2	-	-
		5	0.9	17.2	-	22.1	51.6	8.2	0.45	-	42.37	15.78	3.7	-	-
		10	1.8	16.3	-	22.1	51.6	8.2	0.45	-	41.86	14.92	3.6	-	-
		15	2.7	15.4	-	22.1	51.6	8.2	0.45	-	38.41	12.51	3.2	-	-
		20	3.6	14.5	-	22.1	51.6	8.2	0.45	-	34.87	10.24	2.8	-	-

## 5. Waste Paper/Cardboard Cement Composite

**Figure 5:**
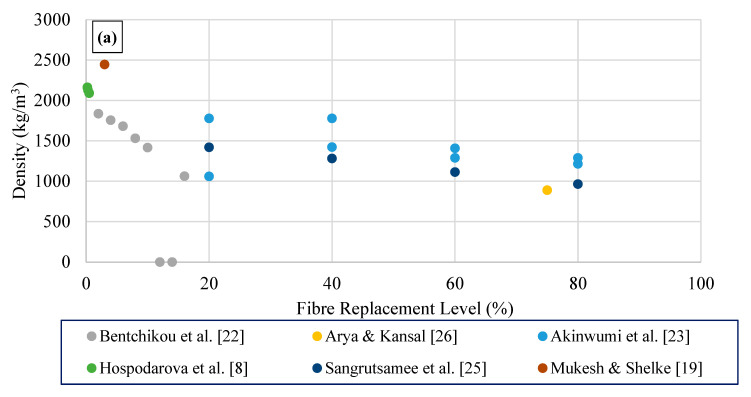

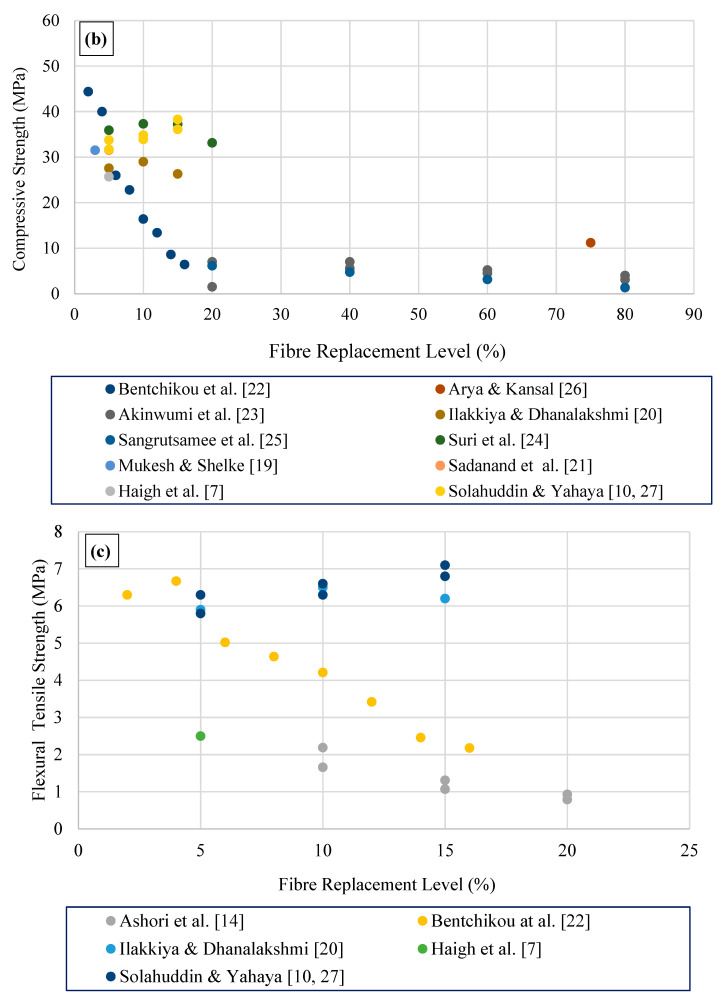

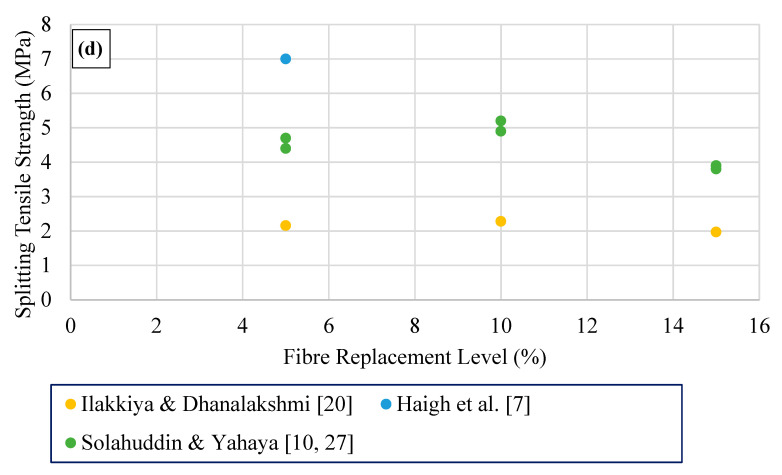
(**a**) Density; (**b**) 28-day compressive strength; (**c**) 28-day flexural tensile strength; and (**d**) 28-day splitting tensile strength variations of waste paper/cardboard cement composite binders.

The mix design and mechanical properties of waste paper-based cement composites are summarised in Table 5 and Figure 5. Contrasting conclusions have been made by different researchers on the effect of waste paper cellulose fibre replacement on mechanical properties. When considering fresh properties (i.e., workability), Mukesh and Shelke [27] reported a 62 mm slump value for 3% replacement of paper fibre in concrete. Ilakkiya and Dhanalakshmi [28] reported addition of paper from 0 to 15% resulted in reducing the slump value from 80 to 72 mm, respectively. Similar observations were reported by Sadanand et al. [29] (85 and 75 mm slump for 5 and 10% waste paper replacement, respectively) for concrete prepared with waste paper fibres.

Cellulosic fibres are more effective lightweight raw material. Addition of cellulosic fibres led to the reduction of density of cement composites, depending on the amount of cellulose fibres as well as the type of cellulose fibres. As the fibre content increases from 0 to 16%, bulk density decreases from 1972 to 1063 kg/m^3^, providing a lightweight construction material [30]. A reduction in density values (approximately 25% reduction) was observed by Akinwumi et al. [31] with the inclusion of waste paper fibres from 20 to 80% in cement composites.

The study by Ashori et al. [22] investigated the reinforcing effect of recycled paper in cement boards manufactured using fibre-to-cement ratios of 10:90, 15:85, and 20:80 by weight. The development of modulus of rupture from 1.5 to 2.2 MPa and modulus of elasticity from 0.5 to 2.5 GPa was observed for 10% replacement of cement by paper fibre (with 5% accelerators) when compared with the control specimens prepared without waste paper cellulose fibres. However, an approximate 44–63% reduction was observed in modulus of rupture (from 2.2 to 0.9 MPa) and modulus of elasticity (from 2.5 to 0.8 GPa) from 10% to 20% addition of waste paper cellulose fibres for cement paste. Bentchikou et al. [30] observed an 85% reduction in compressive strength reduction, from 44.4 to 6.4 MPa with the increment of fibre content from 2 to 16%, respectively. Addition of 2% paper fibre drastically reduced the compressive strength by 21.6 MPa, when compared with the control cement composite specimens prepared without waste paper fibre. Ilakkiya and Dhanalakshmi [28] investigated that compressive strength increased initially with the addition of paper pulp from 5 to 10% of paper fibre (approximately by 1.5 MPa), however it decreased significantly on further addition of paper pulp up to 20% (reduced by 2.7 MPa). The compressive strength values increased to 10% of paper pulp addition. A similar trend was observed by Suri et al. [32], who observed that the 28-day compressive strength increased up to 37.31 MPa with the replacement of waste paper up to 10% and further increments of waste paper up to 20% resulted in a 15% decline (5.8 MPa) in compressive strength.

Furthermore, the addition of 5% recycled cardboard kraft fibre showed 11.7 MPa compressive strength for cement-based composite binders reported by Haigh et al. [15]. Mukesh and Shelke [27] reported 28-day compressive strength of 31.51 MPa with 3% replacement of cement with waste paper fibres. Sangrutsamee et al. [33] investigated that the compressive strength decreased from 6.14 to 1.33 MPa with the addition of a waste paper range from 20 to 80%, respectively.

Flexural strength does not show an incremental behaviour with waste paper/cardboard fibre content, as reported by Ashori et al. [22] and Bentchikou et al. [30]. Bentchikou et al. [30] reported the flexural strength increased from 5.8 to 6.7 MPa between 0 and 4% fibre content, respectively. However, Solahuddin and Yahaya [18] observed incremental flexural strength behaviour (from 6.3 to 7.1 MPa) with waste paper replacement from 5 to 15%, respectively. An incremental trend in splitting tensile strength was reported by researchers [18,28] up to 10% replacement of waste paper content, and further increments of waste paper fibre up to 15% resulted in a 14–25% decline in splitting tensile strength.

**Figure 6:**
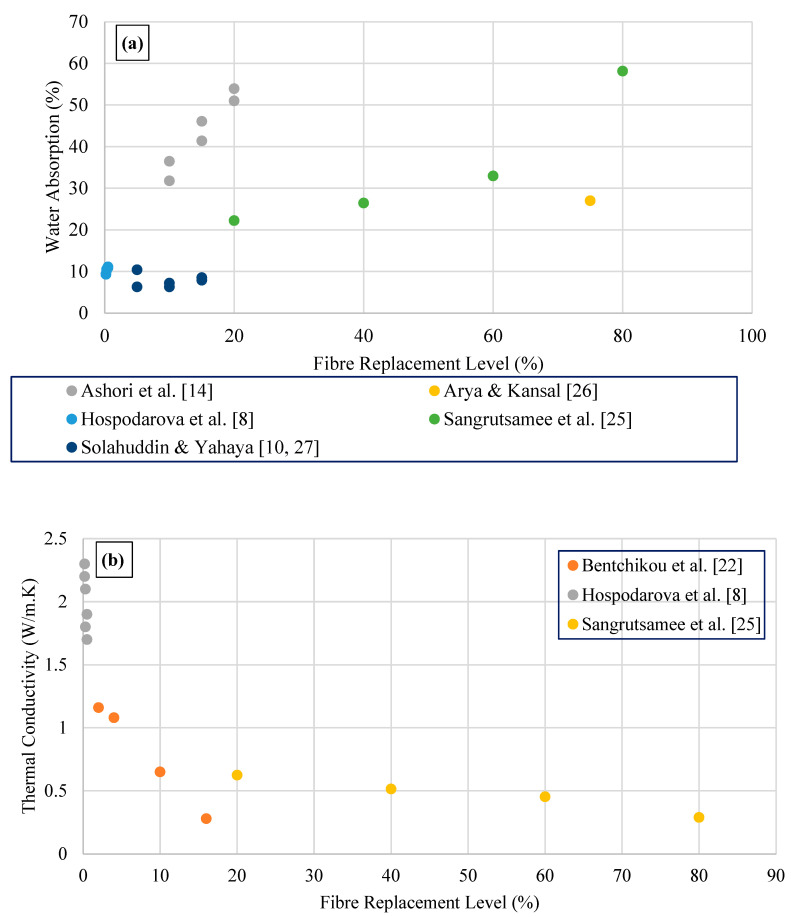
(**a**) Water absorption and (**b**) thermal conductivity variation of waste paper/cardboard cement composite binders.

Water absorption and thermal conductivity variation of waste paper cement composite binders are illustrated in Figure 6. Results reported by Ashori et al. [22] observed that the amount of paper fibre increases the water absorption of the cement composite boards (prepared with paste) increased significantly. It was observed that the sharp increase in water absorption when the paper fibre content in the mix was more than 10% by weight. Sangrutsamee et al. [33] investigated that the incremental water absorption trend from 22.2 to 58.1% with the replacement level of waste paper ranges from 20 to 80%, respectively. Rajput et al. [20] observed 105, 101.6, and 99.3% of water absorption for cement paste bricks prepared with cellulose waste paper with 85, 87, and 89% weight.

Hospodarova et al. [16] observed reduction in thermal conductivity with the addition of paper fibre in cement binders. The results show that better thermal conductivity (values lower by 33.9% in comparison with reference mix (2.70 W/m.K)) were observed for samples (1.78 W/m.K) which were prepared using the 0.5% amount of cellulosic fibres, respectively. Furthermore, these results were compared with thermal conductivity. Using cellulose fibres yield a better thermal insulation behaviour of cementitious matrix, where k = 0.28 W/m.K at 16% fibre content [30].

**Table 5:** Mix design and performance of cellulose waste (waste paper and cardboard) cement-based composite.

Reference	Paper Type	Replacement Level (%)	Waste Cellulose Paper (wt %)	Cement (wt %)	Other (wt %)	Sand (wt %)	Coarse Aggregate (wt %)	Water (wt %)	Water/Cement	Density (kg/m^3^)	Compressive Strength (MPa)	Flexural Strength (MPa)	Splitting Tensile Strength (MPa)	Elastic Modulus (GPa)	Water Absorption (%)	Thermal Conductivity (W/mK)
[22] CaCl_2_	Recycled paper	10	6.4	57.3	1.9	-	-	34.4	0.6	-	-	1.66	-	2.27	36.47	-
		10	6.3	56.6	3.1	-	-	34.0	0.6	-	-	2.19	-	2.45	31.76	-
		15	9.7	55.2	2.0	-	-	33.1	0.6	-	-	1.07	-	1.09	46.08	-
		15	9.6	54.5	3.2	-	-	32.7	0.6	-	-	1.31	-	1.4	41.37	-
		20	13.2	53.0	2.0	-	-	31.8	0.6	-	-	0.79	-	0.65	53.92	-
		20	13.1	52.3	3.2	-	-	31.4	0.6	-	-	0.93	-	0.83	50.98	-
[30]	Recycled paper and cardboard	2	1.5	71.4	-	-	-	27.1	0.38	1836	44.4	6.3	-	-	-	1.16
		4	2.9	69.4	-	-	-	27.7	0.4	1755	40	6.67	-	-	-	1.08
		6	4.3	67.4	-	-	-	28.3	0.42	1681	26	5.02	-	-	-	-
		8	5.4	62.2	-	-	-	32.4	0.52	1531	22.8	4.64	-	-	-	-
		10	6.6	59.8	-	-	-	33.5	0.56	1416	16.4	4.21	-	-	-	0.65
		12	7.9	57.6	-	-	-	34.6	0.6	-	13.4	3.42	-	-	-	-
		14	9.0	55.5	-	-	-	35.5	0.64	-	8.6	2.46	-	-	-	-
		16	10.2	53.5	-	-	-	36.4	0.68	1063	6.4	2.18	-	-	-	0.28
[34]	Paper—Wet pulp	75	60	20	-	20	-	NA	NA	889.3	11.2	-	-	-	27	-
[31]	Newspaper	20	9.1	45.5	-	45.5	-	NA	NA	1422.2	7.0	-	-	-	-	-
		40	16.7	41.7	-	41.7	-	NA	NA	1288.9	5.6	-	-	-	-	-
		60	23.1	38.5	-	38.5	-	NA	NA	1214.8	4.5	-	-	-	-	-
		80	28.6	35.7	-	35.7	-	NA	NA	1060.7	3.1	-	-	-	-	-
	Office Paper	20	9.1	45.5	-	45.5	-	NA	NA	1407.7	1.5	-	-	-	-	-
		40	16.7	41.7	-	41.7	-	NA	NA	1288.9	7.0	-	-	-	-	-
		60	23.1	38.5	-	38.5	-	NA	NA	1125.9	5.2	-	-	-	-	-
		80	28.6	35.7	-	35.7	-	NA	NA	1049.0	4.0	-	-	-	-	-
[16]	Recycled waste paper	0.2	15.0	18.7	56.1	-	-	10.3	0.55	2161	-	-	-	-	9.3	2.3
		0.3	20.9	17.4	52.2	-	-	9.6	0.55	2126.9	-	-	-	-	10.5	2.1
		0.5	30.5	15.3	45.8	-	-	8.4	0.55	2088.8	-	-	-	-	11.1	1.9
		0.2	15.0	18.7	56.1	-	-	10.3	0.55	2162.8	-	-	-	-	9.4	2.2
		0.3	20.9	17.4	52.2	-	-	9.6	0.55	2128.2	-	-	-	-	10.3	1.8
		0.5	30.	15.3	45.8	-	-	8.4	0.55	2091.9	-	-	-	-	10.9	1.7
[28]	Paper pulp	5	-	-	-	-	-	-	-	-	27.57	5.9	2.16	-	-	-
		10	-	-	-	-	-	-	-	-	28.99	6.5	2.28	-	-	-
		15	-	-	-	-	-	-	-	-	26.31	6.2	1.97	-	-	-
[33]	Paper pulp	20	7.0	35.2	-	29.6	-	28.2	0.8	1420.3	6.14	-	-	-	22.22	0.6250
		40	12.0	29.9	-	25.2	-	32.9	1.1	1281.08	4.74	-	-	-	26.44	0.5149
		60	15.6	26.0	-	21.9	-	36.4	1.4	1113.08	3.13	-	-	-	32.96	0.4529
		80	18.4	23.0	-	19.4	-	39.2	1.7	965.36	1.33	-	-	-	58.14	0.2890
[32]	Paper pulp	5	-	-	-	-	-	-	-	-	35.92	-	-	-	-	-
		10	-	-	-	-	-	-	-	-	37.31	-	-	-	-	-
		15	-	-	-	-	-	-	-	-	37.22	-	-	-	-	-
		20									33.15	-	-	-	-	-
[27] Paper pulp		3	1	30.7	1.7	33.3	33.3	NA	NA	2444.4	31.51	-	-	-	-	-
[29]	Paper pulp (newspaper)	5	-	-	-	-	-	-	-	-	25.7	-	-	-	-	-
		10	-	-	-	-	-	-	-	-	22.76	-	-	-	-	-
[15]	Recycled cardboard kraft fibre	5	-	-	-	-	-	-	-	-	11.7	2.5	7	-	-	-
[18,35]	Paper	5	4.2	25.6	-	19.2	38.3	12.8	0.5	-	33.75	6.3	4.4	-	6.3	-
		10	8.0	24.5	-	18.4	36.8	12.3	0.5	-	34.84	6.6	4.9	-	7.2	-
		15	11.5	23.6	-	17.7	35.4	11.8	0.5	-	38.28	7.1	3.8	-	8.5	-
	Cardboard	5	4.2	25.6	-	19.2	38.3	12.8	0.5	-	31.72	5.8	4.7	-	10.4	-
		10	8.0	24.5	-	18.4	36.8	12.3	0.5	-	33.9	6.3	5.2	-	6.3	-
		15	11.5	23.6	-	17.7	35.4	11.8	0.5	-	36.1	6.8	3.9	-	7.9	-

## 6. Discussion

Several factors could lead to adverse effects on the fresh properties (i.e., workability) of paper fibre composites. The amount of paper replacement, physical properties, and the carbon content of the waste paper fibre are the main reasons for the reduction of concrete workability. The water demand becomes larger with an increase in the waste paper fibre content. The paper exhibited a highwater absorption capacity due to its hydrophilic characteristics of the fibre. Paper fibre absorbs a large amount of water and consequently the consistency of the cementitious mixture is greatly reduced, thus resulting in lower workability. Even though there are no reported experimental data for setting time, it is hypothesised that the presence of silica and magnesium led to the improvement of the setting of the cement composite with the addition of waste paper.

The addition of both waste paper and paper sludge has shown both increased and decreased mechanical properties in cement binders. The final properties of cellulose fibre cement composites depend on the fibre, the matrix components, and the manufacturing process. The mechanical performance of the composites depends not only on the matrix and fibre characteristics, but also on the interface properties. Besides the fibre length, the composite toughness is mainly governed by fibre–matrix bonding. A well-balanced interaction between the cement matrix and the fibres which allows fibre debonding and pull-out as well as the stress transfer from the matrix to the fibres is necessary to obtain cellulose cement composites with high toughness. Furthermore, a homogeneous dispersion of the fibres in the matrix, a low porosity of the matrix, and an optimised percentage of fibres is enough to reinforce the material while allowing a continuity of the matrix and are crucial factors to achieve in order to develop composites with well-balanced mechanical properties incorporating waste paper cellulose fibres. A higher amount of cellulose fibre resulted in difficulty of maintaining a homogenous mixture, thus reducing strength properties. In addition, the lower specific gravity of the paper fibre (about 0.45) causes fibres to float on top of the slurry and it creates a lack of homogeneous mixture in the composite so that the top surface of the composite fills with accumulated fibres. This resulted in reduced strength properties in cellulose fibre waste cement base composite binders [22].

**Figure 7:**
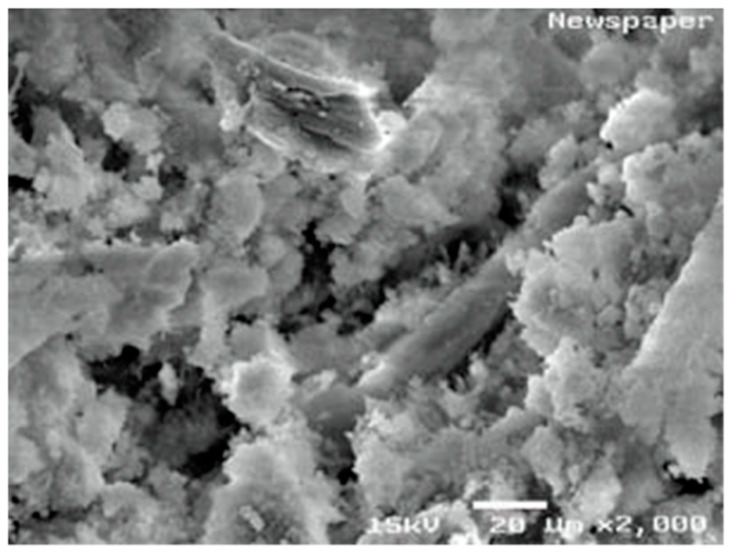
SEM image of cement composite prepared with waste cellulose paper fibre [33].

During the early stages of hydration, the saturation of gel pore water intensifies as C-S-H growth occurs. This changes the result of the capillary pores located near the cellulose fibres. The capillary pores decrease due to the hydrophilic nature of cellulose fibres, leaving an increase of consumed water within the matrix of the composite. This could result with a decrease in porosity as compared to composites containing hydrophobic fibres. However, addition of cellulose fibre could lead to increases in the porosity when compared with the specimens prepared without waste paper cellulose fibres. This is due to the fact that increasing the content of cellulose fibre yields a lot of voids in the specimen. This is hypothesised as one of the reasons that leads to a lower compressive strength and bulk density when compared with conventional PC concrete. The SEM observations by Sangrutsamee et al. [33] (Figure 7) of the interfacial transition zone contrast poor bonding of fibre particles with cement paste. Thus, it results in an increase in tiny voids and porosity in the matrix.

Overall, the flexural strength does not show monotonic behaviour with addition of water paper cellulose fibre content. The flexural strength increases between 0 and 4% fibre content, probably due to the bridging effect of fibres in the matrix. However, beyond 4% replacement, the flexural strength decreases progressively afterwards [30]. This decline behaviour is mainly due to the effect of fibre related to nonuniform dispersion on the matrix and the weakening response following a reduction of cementitious matrix volume proportion. Several authors [30,36,37] reported that, in the fracture process of fibre-reinforced concrete, crack bridging effects induced by fibres can improve resistance to crack propagation and crack opening.

**Figure 8:**
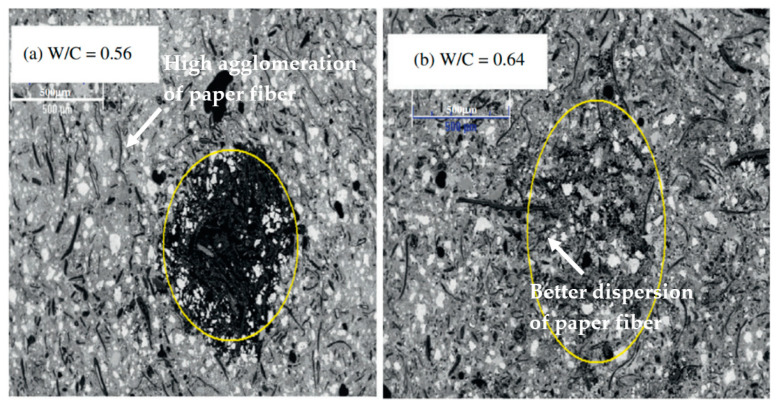
Microstructure of 10% replacement of waste paper fibres [30].

When adding waste paper cellulose fibres to cement, the amount of water needed is increased for compensating the water absorbed by fibres [30]. Microstructure investigation with SEM is shown in Figure 8a for a composite with 10% replacement of waste paper fibres (water-to-cement ratio of 0.56); it illustrates the area in the composite where agglomerations of fibres occur, which is a sign of non-homogeneous dispersion of fibres into the matrix. With the increased water-to-cement ratio of 0.64 (with the addition of water), the microstructure of the composite (Figure 8b) showed a better dispersion of fibres in the matrix with a zone where little agglomeration was found (marked by the circle in Figure 8). The better dispersion of fibres, obtained by adding water, has however an impact on the strength. The strength slightly increases up to water/cement of 0.64 and then it declines with the addition of extra water (water/cement > 0.64) [30]. This is due to the higher voids and porosity due to the higher amount of water content on the cement composites. Even though the effect of fibre dispersion in the matrix improves with the addition of water, higher porosity and voids govern the strength properties beyond an optimum water-to-cement ratio.

Overall, all reported results indicate that the presence of a high amount of waste paper and water paper sludge in the mixture yields high water absorption. This phenomenon is related to the hydrophilic nature of cellulosic fibres absorbing some volume of water due to their microporous structure. Hence, paper fibre absorbs a high amount of water leading to higher water absorption. Furthermore, the chemical properties of the paper type affect the water absorption of the ultimate cement composite binders. Ashori et al. [22] reported that the higher amount of hemicellulose content (17.2%) in waste paper fibres result in a high water absorption rate. Moreover, higher porosity with the addition of paper fibre in cement binders leads to increases in the water absorption capacity and lowers the thermal conductivity of specimens.

## 7. Future Studies

It is highly recommended that future research should be undertaken over longer periods (i.e., beyond a one-year period) to understand the long-term engineering performance. More investigation of the cellulose cement-based materials is needed based on air permeability, water permeability, carbonation, and sulphate and acid attack to ascertain performance with regard to the durability of the waste cellulose paper cement composite binders. Further research is recommended to investigate those in-depth microstructural and pore characteristics to understand the reaction mechanism, issues in the destruction of hemicellulose in an alkaline environment of the current matrix during long-term operation of products prepared with cardboard and paper waste, and other crucial factors that govern the mechanical and durability properties behind the performance of waste paper cellulose fibre cement composite binders. Moreover, numerical predictive modelling and carrying out large-scale tests in the laboratory scale for concrete elements will be a more practical and accurate way to find the behaviour of cellulose cement-based materials. Conducting a detailed analysis of environmental impact assessment and whole life costing of concrete integrated cellulose fibres from raw material preparation stage (cradle) to end of life (grave) stage is recommended for future studies considering circularity of this reclaimed waste into value-added applications.

## 8. Summary and Conclusions

This review presents the research conducted in the field of cement-based composites reinforced with waste paper-based cellulose fibres, focusing on their composition and engineering performance. The summary and the principal conclusions obtained from the review are presented as follows:

- Reduction in density values, i.e., approximately 25% was observed with the inclusion of waste paper fibres from 20 to 80% in cement composites.
- The homogeneous dispersion of the fibres in the matrix was identified as one of the crucial factors to achieve in order to develop composites with well-balanced mechanical properties incorporating waste paper cellulose fibres.
- The dispersion of fibres can be improved by increasing water quantity corresponding to the optimal value (optimum water/cement of 0.64) leading to optimum strength performances (28 MPa compressive strength) of the composite.
- The waste paper cellulose fibre exhibited a high water absorption capacity due to its hydrophilic characteristics and microporous structure, thus leading to lower workability and higher water absorption with the addition of waste cellulose fibre in cement composite binders.
- The capillary pores decrease due to the hydrophilic nature of cellulose fibres, leaving an increase of consumed water within the matrix of the composite. This could result in a decrease in porosity as compared to composites containing hydrophobic fibres.

## Figures and Tables

**Table 1:** Paper and cardboard waste status of Australia [3].

Item	Statistics in 2018–2019
Paper and cardboard usage	235 kg (annually, per person)
Total paper and cardboard waste generation	5.92 million tonnes (office paper 2.06 million tonnes and packaging materials 3.86 million tonnes)
Disposed at landfill	1.9 million tonnes
Recovery rate	60% (3.53 million tonnes)
Export (expected to stop by mid-2024)	0.375 million tonnes
Remaining waste before export	0.49 million tonnes

**Table 2:** Chemical and morphological properties of recycled paper fibres [16,22].

Characteristics	Ashori et al. [22]	Hospodarova et al. [16]
α-cellulose (%)	53.2 ± 2.4	80
Hemicellulose (%)	17.2 ± 1.3	-
Lignin (%)	20.7 ± 1.3	-
Extractives (%)	5.8 ± 0.5	-
Ash (%)	1.6 ± 0.2	20
Fibre length (mm)	0.87 ± 0.42	0.6, 1.2
Fibre width (mm)	24.3 ± 7.6	-
Aspect ratio	37.1 ± 3.2	-
Freeness (CSF)	560	-

**Table 3:** Elemental analysis of paper waste sludge [19,20,23].

	Percentage of Elements (%)												
	O	Ca	Si	Al	Mg	S	Ti	K	Fe	Na	Cu	P	Cl
Recycled paper mill waste [20]	15.8	14.9	60.5	2.1	3.6	1.07	0.15	0.16	0.92	0.22	0.05	0.03	0.41
Paper sludge [23]	CaO	Al_2_O_3_	SiO_2_	K_2_O	SO_3_	Fe_2_O_3_	MgO	TiO2	SrO	ZnO	Cl	Na	K
	79	9	8	1	1	1	-	<1	<1	<1	<1	<1	<1
[19]	46.2	3.6	9	-	-	-	3.3	-	-	-	-	-	-

## Data Availability

Data is contained within the article.
